# Stimuli‐responsive crosslinked nanomedicine for cancer treatment

**DOI:** 10.1002/EXP.20210134

**Published:** 2022-04-21

**Authors:** Xiangdong Xue, Haijing Qu, Yuanpei Li

**Affiliations:** ^1^ School of Pharmacy, Pharm‐X Center Shanghai Jiao Tong University Shanghai China; ^2^ Department of Biochemistry and Molecular Medicine, UC Davis Comprehensive Cancer Center University of California Davis Sacramento California USA

**Keywords:** cancer treatment, crosslinking strategy, drug delivery, stimuli responsiveness

## Abstract

Nanomedicines are attractive paradigms to deliver drugs, contrast agents, immunomodulators, and gene editors for cancer therapy and diagnosis. However, the currently developed nanomedicine suffers from poor serum stability, premature drug release, and lack of responsiveness. Crosslinking strategy can be utilized to overcome these shortcomings by employing stimuli‐responsive chemical bonds to tightly hold the nanostructure and releasing the payloads spatiotemporally in a highly controlled manner. In this Review, we summarize the recently ingenious design of the stimuli‐responsive crosslinked nanomedicines (SCN) in the field of cancer treatment and their advances in circumventing the drawbacks of the conventional drug delivery system. We classify the SCNs into three categories based on the crosslinking strategies, including built‐in, on‐surface, and inter‐particle crosslinking nanomedicines. Thanks to the stimuli‐responsive crosslinkages, SCNs are capable of keeping robust stability during systemic circulation. They also respond to the particular tumoral conditions to experience a series of dynamic changes, such as the changes in size, surface charge, targeting moieties, integrity, and imaging signals. These characteristics allow them to efficiently overcome different biological barriers and substantially improve the drug delivery efficiency, tumor‐targeting ability, and imaging sensitivities. With the examples discussed, we envision that our perspectives can inspire more attempts to engineer intelligent nanomedicine to achieve effective cancer therapy and diagnosis.

## Graphical abstract

We classify the stimuli‐responsive crosslinked nanomedicines (SCNs) into three categories based on the crosslinking strategies, including built‐in, on‐surface, and inter‐particle crosslinking nanomedicines. These SCNs can respond to particular tumoral stimuli and go through a series of dynamic changes to circumvent different biological barriers, and subsequently improve anti‐cancer efficacy and imaging sensitivity and specificity.

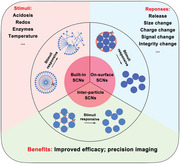

## 1 INTRODUCTION

Nanomedicine has emerged as a powerful tool to overcome the shortcomings associated with conventional cancer treatments.^[^ ^1^ ^]^ Thanks to sophisticated nanotechnology, nanomedicines enable the orchestration of a myriad of excellent features, such as multifunctionality, controllability, and tumor selectivity. These features allow them to be applied to different diseases, while some nanomedicines have already shown great promise in clinical practice. Nanomedicines bring many benefits to conventional therapeutics and contrast agents, such as improved water solubility, prolonged blood circulation, desirable biodistributions, better efficacy/accuracy, biocompatibility. Nanomedicines enable the delivery of multiple drugs to the tumor sites simultaneously, which can make up the difference in the pharmacokinetic (PK) profiles of the drugs, and therefore, realize a “1 + 1 > 2” effect.^[^ ^2^ ^]^ Moreover, theranostic nanomedicines concomitantly carry therapeutic and diagnostic agents to realize anti‐tumor effect coupled with imaging capabilities. The imaging capacities, such as near‐infrared fluorescence imaging (NIRFI),^[^ ^3^ ^]^ magnetic resonance imaging (MRI),^[^ ^4^ ^]^ positron emission tomography (PET)^[^ ^5^ ^]^ and photoacoustic imaging,^[^ ^6^ ^]^ etc. provide valuable details of the biodistributions and therapeutic outcomes to make nanomedicine‐based treatments more feasible.^[^ ^7^ ^]^ Nanomedicines with spatiotemporal controllability can unload the payloads in desirable manners, like targeted release, controlled release, and sustained release.^[^ ^8^ ^]^ The payload release can be either triggered by internal stimuli, such as the acidic pH,^[^ ^8^, ^9^ ^]^ high redox pressure,^[^ ^10^ ^]^ and tumor‐specific enzymes^[^ ^11^ ^]^ that exist in tumor microenvironment (TME), or external stimuli, including laser irradiation,^[^ ^8^, ^12^ ^]^ magnetic field,^[^ ^13^ ^]^ and ultrasound.^[^ ^14^ ^]^ Nanomedicines also exhibit excellent tumor selectivity by taking advantage of the enhanced permeability and retention (EPR) effect^[^ ^15^ ^]^ or transcytosis,^[^ ^16^ ^]^ which can minimize the off‐target toxicity of the therapeutics and undesirable background noise of imaging agents. Taking all these unique features together, nanomedicines can significantly enhance the therapeutic index of traditional therapeutics, and improve imaging sensitivity and specificity.

Although intriguing to cancer treatment, nanomedicines still face multiple challenges in the drug delivery process to tumors.^[^ ^17^ ^]^ Shen's group summarized these challenges as a 5‐step CAPIR cascade^[^ ^18^ ^]^ which includes circulation (C), accumulation (A), penetration (P), internalization (I) and drug release (R). The delivery efficiency will plummet if the nanomedicine does not perform well in one of these steps, as these five steps are in a cascade system. For instance, Doxil® can extensively improve the blood circulation and tumor site accumulation of doxorubicin (DOX); however, it only shows marginal efficacy improvement to DOX due to the ineffective tumor penetration.^[^ ^19^ ^]^ Nanomedicines, such as micelles, liposomes, and polymeric nanoparticles, are all spontaneous self‐assemblies made by amphiphilic building blocks. The self‐assembly process follows the thermo‐dynamic rules, and is mainly driven by the noncovalent interactions, like hydrophobic force, hydrogen bond, van der Waals, electrostatic interaction, etc. In the CAPIR cascade, serum stability is the first challenge when the nanomedicines are administrated intravenously. In the bloodstream, many adverse conditions, such as blood sheer force, opsonization,^[^ ^20^ ^]^ and protein corona,^[^ ^21^ ^]^ can damage the nanostructure and lead to the premature leakage of the payloads. Tethering drugs to nanoparticles via reversible chemical bonds^[^ ^22^ ^]^ may be a feasible way to prevent premature drug release and realize controllable drug release; however, nanomedicines still face the risk of structural collapse during blood circulation. The nanomedicines can accumulate in the tumor sites either by passive or active targeting. However, these targeting effects may no longer be valid if the nanostructure collapses before arriving at the destination. Even the nanomedicine arrives at the tumor site, they still need to penetrate the cell membrane to be internalized into tumor cells. In the final stage, drug release is another challenge that affects the efficacy. Stimuli‐responsive crosslinked nanomedicines (SCNs) show great potential to overcome the biological barriers during cancer treatment.^[^ ^1a^ ^]^ The SCNs exhibit much better anti‐tumor efficacy than the non‐crosslinked counterparts, as the robust stability gifted by crosslinking strategy guaranteed long blood circulation and effective drug release of the drugs in the tumor site. The crosslinking strategy can tightly hold the nanostructure during blood circulation and prevent the drugs from being prematurely released, thus substantially improving PKs and drug delivery efficiency. Once arrive at the tumor site, the crosslinkers can be broken down by particular stimulation and readily release the payloads.

**SCHEME 1:**
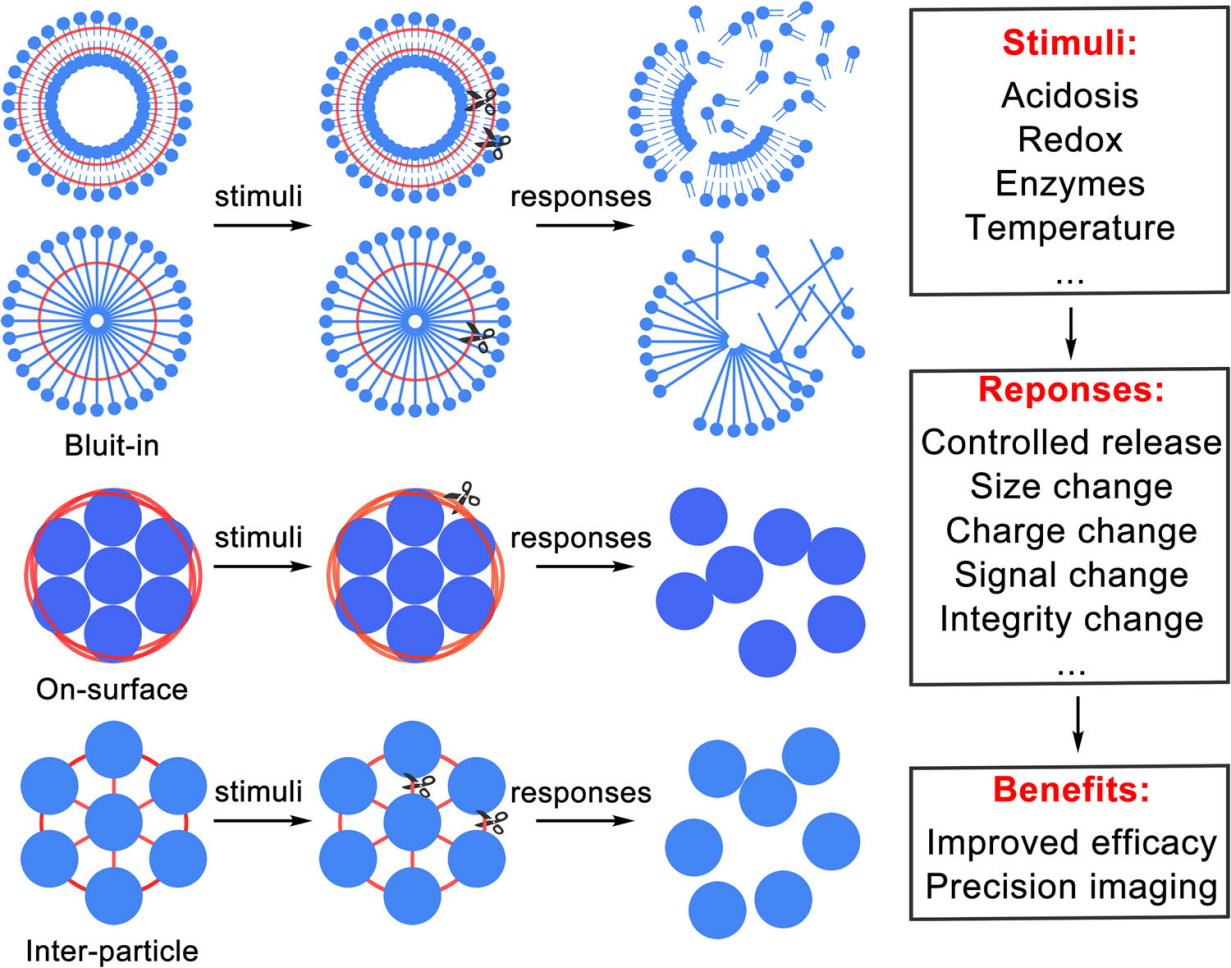
The stimuli‐responsive crosslinking nanomedicine (SCN) is constructed by different strategies. This review classifies the SCN into three categories, including these constructed with built‐in, on‐surface, and inter‐particle crosslinking strategies. The SCN can minimize premature payloads release and exhibit robust stability for long blood circulation and controllable drug release due to responsive crosslinkers. Once the SCN meets the specific stimuli, such as acidic pH, redox pressure, tumor‐abundant enzyme, and hypoxia, a series of responses, such as payload release and changes in size, surface charge, imaging signals, structural integrity, etc. will be triggered. These responses can circumvent the biological barriers during the drug delivery process, bring substantial benefits, such as improved anti‐tumor efficacy and imaging sensitivity, to the cancer treatments. The red ribbons stand for crosslinkers; the scissors are the specific stimuli in TME

In this review, we focused on the recently ingenious design of the SCNs in the field of cancer treatment and their advances in circumventing the drawbacks of the conventional drug delivery system. We summarize the crosslinking strategies into three categories, including built‐in, on‐surface, inter‐particle crosslinking strategies. As shown in Scheme 1, the built‐in crosslinking strategy is realized by embedding the reversible chemical bond in the building blocks of the SCN; the on‐surface crosslinking strategy is made by holding the multiparticle assembly together with reversible linkers on the surface; the inter‐particle crosslinking strategy employs linkers with a reversible chemical bond to crosslink multiple nanoparticles to form relatively larger SCN. These strategies endow the SCN with robust serum stability, sequential responsiveness, high controllability, and tumor selectivity. The crosslinkers prevent the payloads from being prematurely leaked and responsively release them upon a specific stimulation, such as acidic pH, tumor‐specific enzyme, high redox pressure, hypoxia, and oxidative stress. The crosslinkers lock the nanomedicine in a stable status and sequentially respond to the stimulations to trigger corresponding transformations regarding different factors, such as particle size, surface charge, shape, surface ligands, and integrity. Owing to these unique features, the SCN overcomes multiple biological barriers during the delivery process and enables effective cancer therapy and precise cancer diagnosis. We hope the perspectives of SCN can inspire more attempts to develop innovative and precise nanomedicine which can primarily improve cancer treatment in the clinic.

## 2 BUILT‐IN CROSSLINKING STRATEGY

The built‐in crosslinking strategy is the most commonly used method to develop SCNs. It offers a feasible approach to constructing nanostructures because the reversible chemical bonds used as crosslinkers can be directly integrated into the building blocks of nanomedicine. The building blocks self‐assemble into nanostructure, in which the reversible chemical bond can be formed between different blocks to tightly hold the nanostructure as a whole. This strategy has been introduced to construct different SCNs, such as those based on micelles, polymeric nanoparticles, and liposomes. The crosslinked nanomedicines exhibit robust serum stability and stimuli‐responsive drug release, thus extensively improving the drug efficacy and imaging accuracy. In this section, we classified these SCNs according to their responsiveness and reversible chemical bonds.

### 2.1 Built‐in SCNs constructed by disulfide crosslinkeages

The intracellular redox equilibrium is maintained by glutathione (GSH) expression and the intracellular reactive oxygen/nitrogen species. Since the metabolism of the tumor cells is outrageous, the redox imbalance was incurred by tumoral activities. It has been extensively reported that most tumor cells are dominated by overexpressed GSH, which leads to much higher intracellular redox pressure than normal cells.^[^ ^23^ ^]^ Therefore, disulfide‐crosslinked nanomedicine draws much attention to improving tumor specificity, as the high concentration of the GSH in the tumor cells can effectively break the disulfide bond and lead to controllable drug release. The disulfide bonds can be introduced by different approaches, including i) oxidization of free thiol groups^[^ ^24^ ^]^; ii) introducing linkers that contain disulfide bonds, such as 3,3′‐dithiobis (sulfosuccinimidyl propionate)^[^ ^25^ ^]^ and cystamine^[^ ^26^ ^]^; and iii) oxidization of lipoic acid.^[^ ^27^ ^]^ The disulfide crosslinked nanomedicines are generally made by two steps: First, nanomedicine without crosslinkers was assembled by building blocks; Then, the disulfide bonds are generated either by introducing the disulfide linkers directly or oxidizing the thiol moieties to the non‐crosslinked nanomedicine.

**FIGURE 1:**
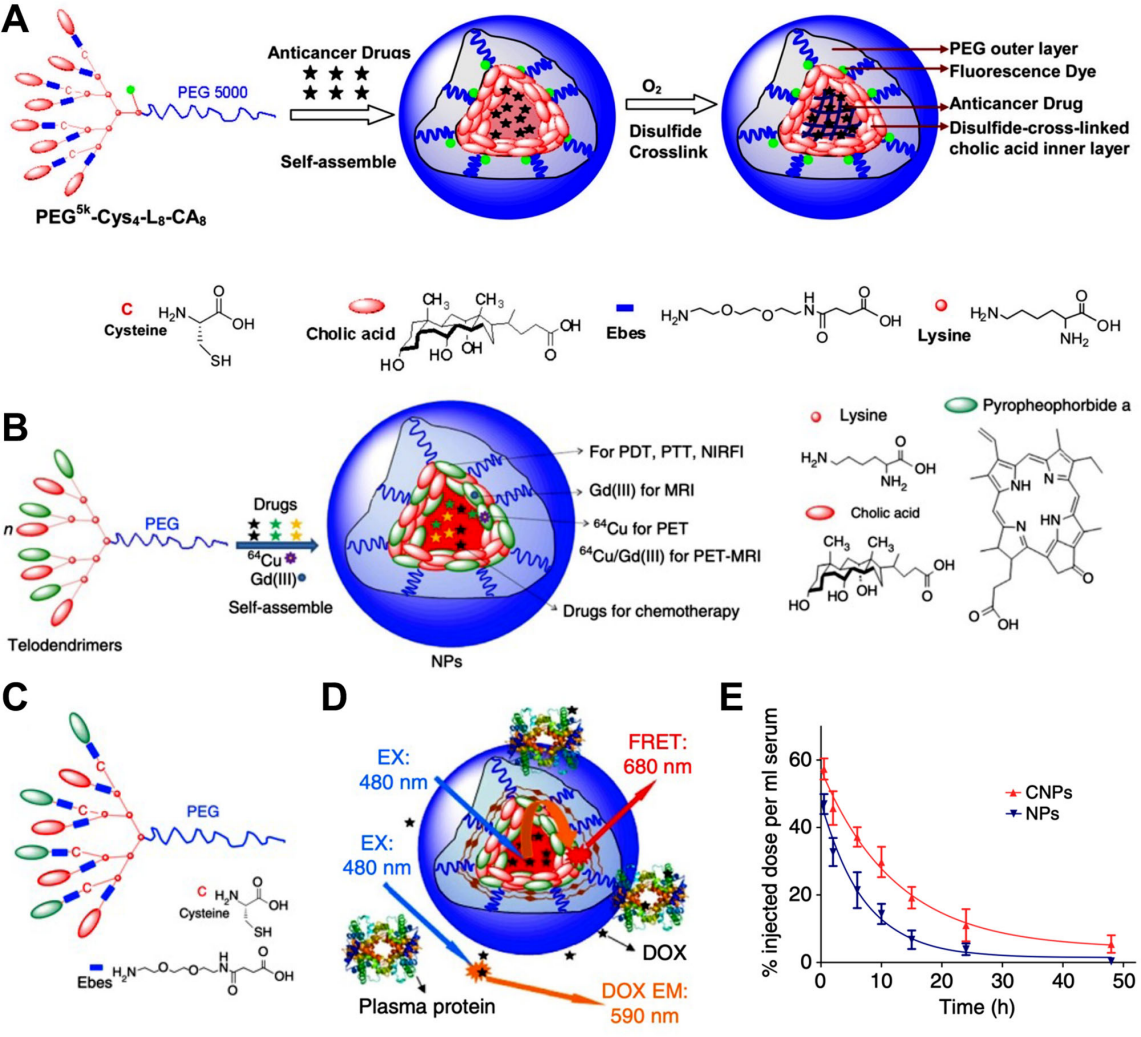
The disulfide crosslinked telodendrimer‐based nanomedicine. (A) The disulfide crosslinked SCN was assembled by thiol‐contained telodendrimers, then oxidized to form inter‐telodendrimer disulfide crosslinkers. Reproduced with permission.^[^ ^24g^ ^]^ Copyright 2014, Elsevier. (B–E) The disulfide crosslinked nanoporphyrin with multifunctionalities. (B) The porphyrin‐telodendrimer based nanomedicine with multiple functionalities. (C) The porphyrin‐telodendrimer with built‐in cysteine for disulfide bond formation. (D) The disulfide crosslinked porphyrin‐telodendrimer based nanomedicine. (E) The pharmacokinetic of the crosslinked porphyrin‐telodendrimer based nanomedicine (CNPs) and the non‐crosslinked counterpart. Adapted with permission.^[^ ^24h^ ^]^ Copyright 2014, Nature Publishing Group

For example, Lam lab developed a polymer with a hydrophilic PEG tail and a hydrophobic dendritic head, which was named as telodendrimer. The telodendrimer exhibits excellent drug‐loading capacities, especially for the highly hydrophobic drug, such as paclitaxel,^[^ ^28^ ^]^ SN‐38.^[^ ^29^ ^]^ To achieve robust serum stability and controllable drug release capacities, disulfide crosslinked nanomedicine was developed by synthesizing thiol‐contained telodendrimer and assembling them into micelles, then oxidizing the thiol groups to form the crosslinkers between telodendrimers. The disulfide crosslinked nanomedicine brings many benefits to cancer therapy and diagnosis, such as controlled structure integrity, improved delivery efficiency, and imaging precision.^[^ ^24g^ ^]^ As shown in Figure 1A, the micelles were first assembled by the telodendrimer, then oxidized to form the disulfide bonds. This disulfide crosslinked micelle (DCM) can load different payloads, such as anticancer drugs and fluorescence dyes, to execute the anticancer efficacy and diagnostic functions concomitantly. The DCM showed robust stability in the physiological condition but quickly released the payloads in the GSH. The DCM showed much better PKs and exhibited higher efficacy to the non‐crosslinked counterpart. The disulfide crosslinker can also improve the self‐assembly of the telodendrimer, and further realize more functionalities. As shown in Figure 1B, Dr. Lam lab replaced the cholic acids partially in the telodendrimer with porphyrin derivatives (pyropheophobride a) to make nanoporphyrin with multiple imaging and therapeutic functionalities.^[^ ^24h^ ^]^ Then, they introduced cysteines to the porphyrin‐telodendrimer to form disulfide crosslinkages (Figure 1C). The disulfide crosslinked nanoporphyrin (Figure 1D) showed much better stability than the non‐crosslinked one and can release the drugs responsively in the cells. Due to the robust stability, the crosslinked nanoporphyrin showed prolonged blood circulation time than the non‐crosslinked counterpart (Figure 1E). The nanoporphyrin exhibited “all‐in‐one” imaging capacities, such as MRI, PET, NIRFI, and showed synergistic therapeutic effect between porphyrin‐mediated phototherapy and DOX‐mediate chemotherapy.

**FIGURE 2:**
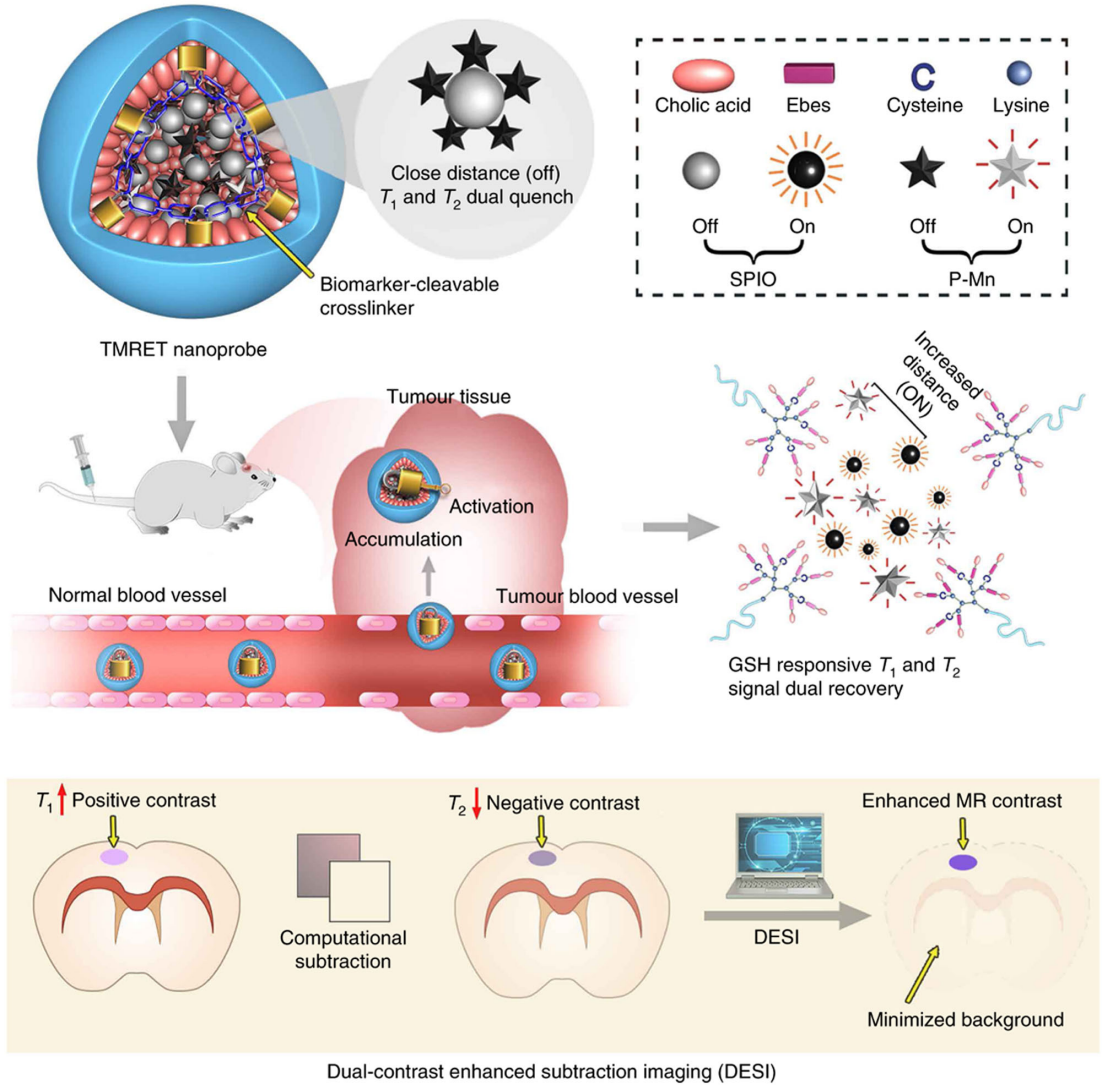
Disulfide crosslinked nanomedicine with tunable T_1_&T_2_ MR signals for imaging of small tumors. GSH can destroy the integrity of nanomedicine and trigger the transformation of nanostructure integrity and tune the subsequent MRI signal for precise cancer diagnosis. Reprinted with permission.^[^ ^24i^ ^]^ Copyright 2020, Nature Publishing Group

MRI provides exquisite details of the lesion with anatomical and functional information, which has been widely used in the clinic as imaging technology for cancer diagnosis.^[^ ^30^ ^]^ To improve the imaging capability of MRI, our group has developed a novel two‐way magnetic resonance energy transfer (TMRET) nanoprobe based on the built‐in crosslinking strategy.^[^ ^24i^ ^]^ As shown in Figure 2, T_1_ contrast agent (pheophorbide a‐manganese chelator, P‐Mn) and T_2_ contrast agent (superparamagnetic iron oxide nanoparticle, SPIO) were co‐encapsulated in the disulfide crosslinked telodendrimer‐based nanomedicine, in which the T_1_ and T_2_ contrast agents were constraint tightly in the hydrophobic core, leading to a dual‐signal quenching phenomenon. The disulfide crosslinkers make the TMRET nanoprobe stable in the blood and prevent the premature release of the T_1_ and T_2_ contrast agents. This results in reduced undesirable background MRI signals. Once the TMRET nanoprobe reached the tumor site, the high redox potential in TME broke down the micelles and released the T_1_ and T_2_ contrast agents, making the interactions between the T_1_ and T_2_ signals invalid, the T_1_ and T_2_ signals thus recovered. With the built‐in crosslinking strategy, the TMRET nanoprobe showed excellent tumor selectivity and specificity. TMRET probe lit up the tumor site but kept silent at normal tissue, which can substantially improve its capability of tumor diagnosis. Besides, we tailored a post‐processing technology for TMRET, named dual‐contrast enhanced subtraction imaging (DESI). DESI technology employed a customized algorithm that accurately subtracts the negative T_2_ images from the positive T_1_ images. The DESI process extensively dimmed the background and enhanced the signal at the tumor site, and therefore further increased the contrast between the tumor site and normal tissue. Combined with our TMRET nanoprobe and DESI technology, this imaging platform can precisely diagnose an intracranial tumor on a mouse model, the tumor size can be as small as 0.75 mm^3^ with a tumor‐to‐normal tissue ratio (TNR) of 12. In this cancer imaging platform, the built‐in crosslinking strategy endows the probe with robust stability, high tumor selectivity, tunable T_1_&T_2_ signal that the DESI technology depends upon.

**FIGURE 3:**
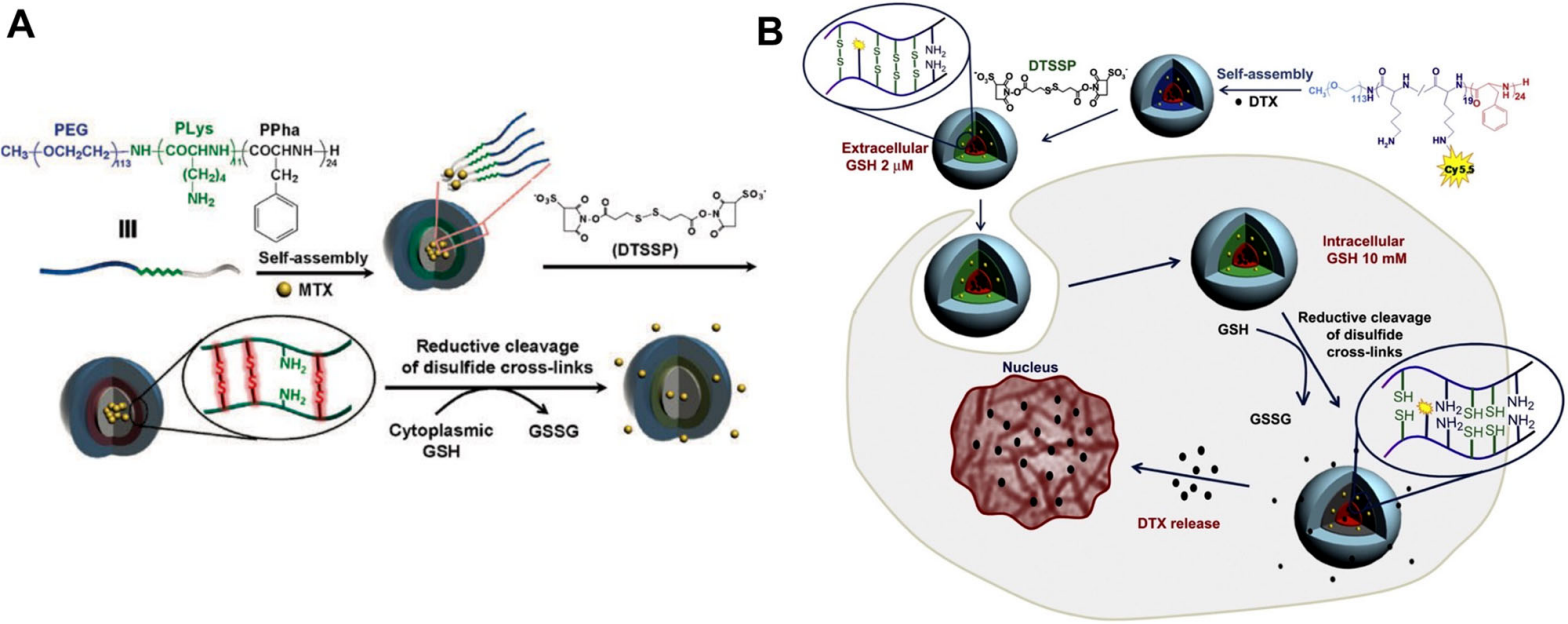
Disulfide crosslinked nanomedicine constructed by post‐modification of the crosslinker (DTSSP). (A) Reprinted with permission.^[^ ^25b^ ^]^ Copyright 2008, Royal Society of Chemistry. (B) Reprinted with permission.^[^ ^25c^ ^]^ Copyright 2012, Elsevier

Active chemicals containing disulfide bond, such as 3,3′‐dithiobis (sulfosuccinimidyl propionate) (DSSTP), cystamine and *N*,*N*‐(ethane‐1,2‐diyl)bis(3‐(pyridine‐2‐disulfanyl)propanamide) (EDPY), etc.,^[^ ^31^ ^]^ are good choices to build disulfide crosslinked nanomedicine. These linkers are introduced to the non‐crosslinked nanomedicine by conjugating with the active moieties in the building blocks. This method does not need oxidation. As shown in Figure 3A, Lee group synthesized an ABC triblock copolymer, poly(ethylene glycol)‐*b*‐poly(l‐lysine)‐*b*‐poly(l‐phenylalanine) (PEG‐*b*‐PLys‐*b*‐PPha).^[^ ^25b^ ^]^ The copolymer can co‐assemble with methotrexate into polymeric nanoparticles. Then, the disulfide‐contained linker, DTSSP, was introduced to crosslink the nanomedicine by reacting with the amine groups on the copolymers. The resultant crosslinked nanomedicine showed GSH responsive drug‐releasing pattern and anti‐lung adenocarcinoma efficacy at the cellular level. Lee group also employed the same procedure to develop a DTX‐loaded disulfide crosslinked micelle by using DSSTP as a crosslinker (Figure 3B).^[^ ^25c^ ^]^ The micelles exhibited better stability than non‐crosslinked counterparts and effectively prevented the premature DTX release. Under the stimulation of GSH, the crosslinked micelles can effectively release the drug. The crosslinked nanomedicine also showed significantly better tumor accumulation and anti‐tumor efficacy on human breast tumor‐bearing mice.

**FIGURE 4:**
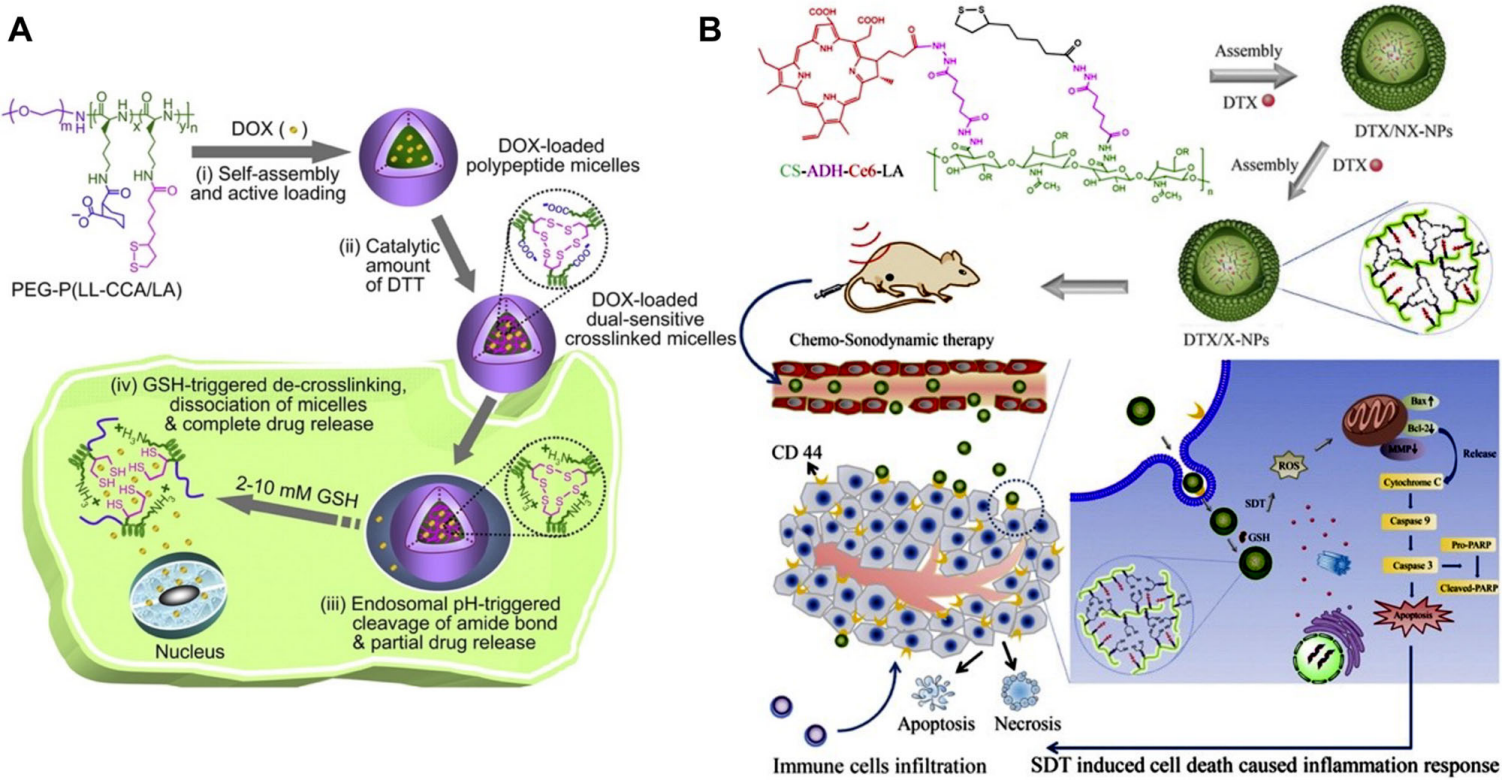
Disulfide crosslinked nanomedicine synthesized by oxidizing the lipoic acid moieties. (A) Multifunctional dextran‐based nanoparticle crosslinked by disulfide bond for cancer chemotherapy. Reprinted with permission.^[^ ^27a^ ^]^ Copyright 2013, Elsevier. (B) Disulfide crosslinked nanomedicine with the combinatorial therapy of chemo‐sonodynamic and immunotherapy for cancer treatments. Reprinted with permission.^[^ ^27b^ ^]^ Copyright 2018, Elsevier

Lipoic acids can be oxidized to form an intermolecular disulfide bond, which has been extensively employed to synthesize building blocks that can form disulfide crosslinkers.^[^ ^27c^ ^]^ Lipoic acids can be introduced into the polymers as the thiol group provider. Upon assembling into polymeric nanoparticles, a catalytic amount of dithiothreitol (DTT) oxidizes the intramolecular disulfide bond to an intermolecular bond, thus crosslinking the polymeric nanoparticles to form more stable nanostructures during blood circulation. In Figure 4A, Zhong et al.^[^ ^27a^ ^]^ synthesized a lipoic acid (LA) and cis‐1,2‐cyclohexanedicarboxylic acid (CCA) decorated poly(ethylene glycol)‐*b*‐poly(l‐lysine) (PEG‐P(LL‐CCA/LA)) block copolymers and co‐assembled DOX to form DOX‐loaded polymeric nanomedicine. A catalytic amount of DTT was used to crosslink the nanomedicine with disulfide bonds. The crosslinked nanomedicine showed high stability in the neutral pH and absence of the GSH. It can readily release the DOX to execute the chemotherapy as the high intracellular concentration of GSH effectively broke the disulfide bond. Zhai et al. synthesized a chitosan‐based polymer with Chlorin e6 (Ce6) and lipoic acid moieties in the structure (Figure 4B).^[^ ^27b^ ^]^ The polymers were co‐assembled with docetaxel into nanomedicine which can be crosslinked by DTT oxidization. The disulfide crosslinked nanomedicine possessed monodispersive size distribution, stability in physiological conditions and showed improved cellular uptaken behaviors and controllable drug release at the presence of the GSH. The nanomedicine efficiently delivered the Ce6 and docetaxel to the melanoma cells and led to effective sonodynamic therapy and chemotherapy mediated by Ce6 and docetaxel, respectively.

### 2.2 Built‐in SCNs constructed by pH‐sensitive crosslinking strategy

The pH‐responsive corsslinkages are the most widely used strategy to design the SCNs, compared to the crosslinkers built with other stimuli. The pH‐sensitive nanomedicines have been extensively developed to achieve effective drug delivery by taking advantage of the pH differences in our body. The gastrointestinal tract showed a wide pH gradient at the organ level that can meet the controllable release of the orally administrated drug. The acidic pH in the TME is also used to stimulate drug release. The extracellular pH in TME is between 6.5 to 6.8 due to the “Warburg effect.”^[^ ^32^ ^]^ This slightly lower pH can be employed to release the drugs taking effect in the TME. At the intracellular level, the acidification of the endsome and their transition to lysosome also provide ideal low pH (∼5.0) for the controllable drug release. So far, the lysosomal pH is one of the most common targets for intracellular drug delivery, as most anti‐tumor drugs directly impact the tumor cells.

**FIGURE 5:**
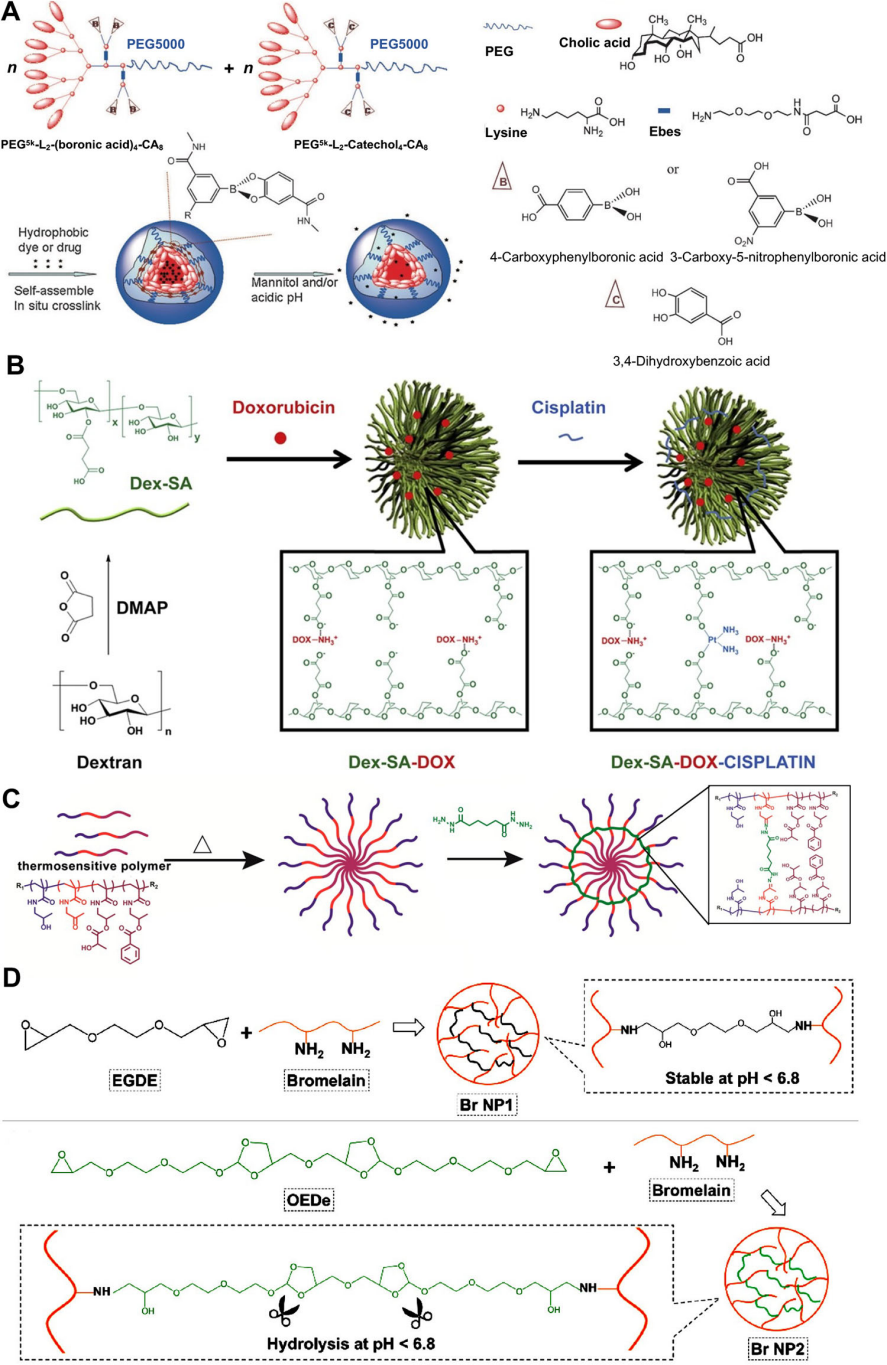
pH‐sensitive crosslinked nanomedicines. (A) Boronic ester crosslinked nanomedicine. Adapted with permission.^[^ ^40^ ^]^ Copyright 2014, Wiley‐VCH. (B) Ester bond crosslinked nanomedicine; Reprinted with permission.^[^ ^41^ ^]^ Copyright 2014, Elsevier. (C) Hydrazone bond crosslinked nanomedicine; Adapted with permission.^[^ ^42^ ^]^ Copyright 2015, American Chemical Society. (D) Orthoester bond crosslinked nanomedicine. Adapted with permission.^[^ ^43a^ ^]^ Copyright 2020, Elsevier

As aforementioned, pH difference is one of the most essential physiological conditions in our body. The pH‐sensitive crosslinkers are extensively explored in drug delivery. They can be used to prevent premature drug release and effectively release the drugs either in extracellular or intracellular pH abnormality that tumor developed. To design pH‐responsive nanomedicine with built‐in crosslinkers, various pH‐sensitive covalent chemical bonds, such as orthoester,^[^ ^33^ ^]^ ester,^[^ ^34^ ^]^ hydrazone,^[^ ^8^, ^36^ ^]^ cis‐aconityl,^[^ ^36^ ^]^ ketal,^[^ ^37^ ^]^ electrostatic force,^[^ ^3^, ^39^ ^]^ boronic ester,^[^ ^39^ ^]^ etc., are embedded into the building blocks. Lam lab has developed an acidic pH‐responsive nanomedicine by introducing the boronic ester to the backbone of the telodendrimer.^[^ ^40^ ^]^ As in Figure 5A, two distinct telodendrimers were synthesized with embedded boronic acid and catechol moieties, respectively. These two telodendrimers were mixed and co‐assembled into polymeric nanomedicine (BCM) with boronate crosslinkers. The BCM showed powerful encapsulation capacity, which can load either the hydrophobic dyes or drugs. Besides acidic pH, the BCM can be broken by mannitols, as the cis‐diols on the mannitol can competitively react with the boronic acid. The BCM exhibited excellent stimuli‐responsive drug release and better PKs than the non‐crosslinked counterpart. Chen group developed a pH‐sensitive crosslinked nanomedicine by using cisplatin as the crosslinker.^[^ ^41^ ^]^ As shown in Figure 5B, they firstly decorated the dextran with succinic acid to introduce the carboxylic acid groups to the backbone of the dextran. The decorated dextran self‐assembled and loaded DOX by electrostatic interaction. Then, the cisplatin was introduced by forming the pH‐reliable ester bond. The resultant nanomedicine showed much better PKs and tumor accumulation than the non‐crosslinked counterparts. Upon stimulated with acidic pH, the nanomedicine can concomitantly release both DOX and cisplatin, and therefore, exhibit highly effective anti‐tumor efficacy on a subcutaneous lung cancer mouse model. Hennink et al.^[^ ^42^ ^]^ synthesized a triblock thermosensitive polymer p(HPMAm)‐*b*‐p(AMPO)‐*b*‐p(HPMAm‐Bz‐*co*‐HPMAm‐Lac). As shown in Figure 5C, the triblock copolymers self‐assembled into polymeric micelles and were crosslinked by adipic acid dihydrazide which can react with the ketone groups on the p(AMPO) to form the hydrazone bond. The crosslinked nanomedicine showed a high drug loading capacity to paclitaxel (∼29%). The crosslinkers can tightly hold the loaded drugs at neutral pH, and responsively release them when stimulated by the acidic pH at 5 in lysosomes. Orthoester is a functional group with three alkoxy groups attached to one carbon atom, which can be readily hydrolyzed in mild aqueous acid to form esters. The chemicals contained in orthoester are frequently employed to build SCN with pH‐responsive crosslinkers.^[^ ^43^ ^]^ Taken one as an example, Tang group linked two bromelains (Br) by a linker containing an orthoester bond (EGDE) and assembled the linked bromelain to crosslinked nanomedicine (Br NP2) with pH responsiveness (Figure 5D).^[^ ^43a^ ^]^ The Br NP2 can effectively load DOX and deliver it to the tumor cells. The Br NP2 releases 86% DOX with 120 h when stimulated by acidic pH (5.5). The function of Br on Br NP2 was not affected by the formation of the nanostructure. It can destroy the dense extracellular matrix and elicit a synergistic anti‐cancer effect with DOX. The tumor growth inhibition of the Br NP2 reached 62.5%, which was much more effective than the crosslinked nanomedicine (Br NP1) with no pH responsiveness, demonstrating that the pH‐responsive crosslinkers were essential to the drug delivery and intracellular drug release.

### 2.3 Built‐in SCNs constructed by metal‐based crosslinking strategy

**FIGURE 6:**
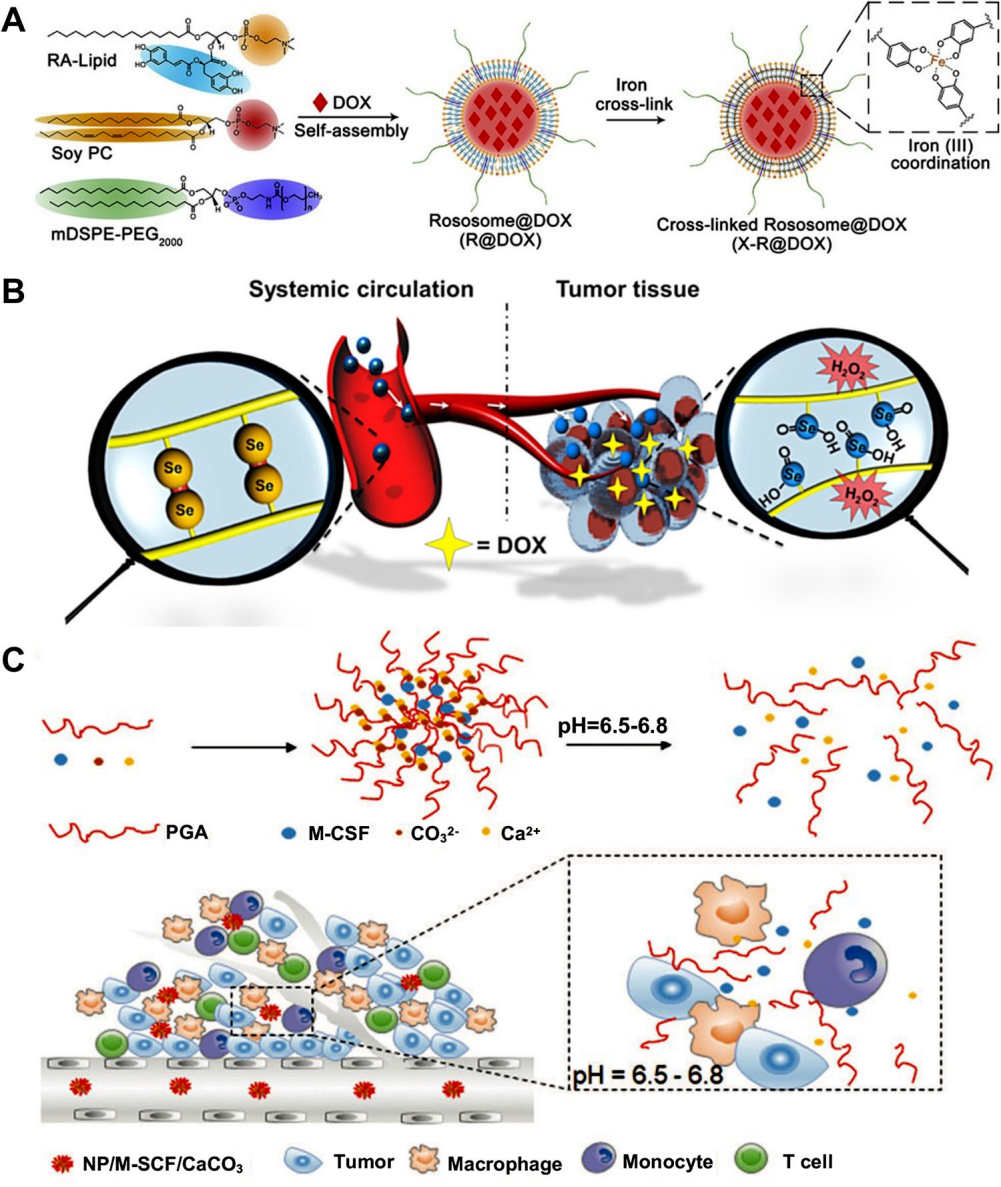
Ion‐based reversible chemistry for the construction of SCNs. (A) Iron‐crosslinked liposome. Reprinted with permission.^[^ ^45^ ^]^ Copyright 2021, Elsevier. (B) Diselenide bond crosslinked nanomedicine. Reprinted with permission.^[^ ^47^ ^]^ Copyright 2016, Elsevier. (C) Calcium crosslinked nanomedicine. Reprinted with permission.^[^ ^48^ ^]^ Copyright 2019, Royal Society of Chemistry

Metal ions can coordinate organic ligands to form stable chemical structures, and such reactions have been widely used to construct metal–organic frameworks (MOFs).^[^ ^44^ ^]^ Similar to the metal–organic coordination in MOF, the metal ions were also employed to build SCNs. As shown in Figure 6A, our group conjugated rosmarinic acid that contained two catechols to the amphiphilic lipid and coassembled them with soy PC to form a liposome.^[^ ^45^ ^]^ The liposomal structure was further crosslinked by incubating with ferric iron that can coordinate with catechols. The crosslinked nanomedicine exhibited robust stability both in vitro and in vivo. Compared to the non‐crosslinked liposome, the crosslinked one can bear the high concentration of the detergent (sodium dodecyl sulfate, SDS) and extensively prolong the blood circulation time, which avoids the premature drug release during the blood circulation. The pH‐reliable crosslinkers made the liposome responsively release the DOX in lysosome pH and exhibit a synergistic anti‐tumor effect with rosmarinic acid. The iron‐catechol coordination was also employed to build crosslinked hydrogel with better strength, stiffness, toughness, and self‐healing abilities.^[^ ^46^ ^]^

Two adjacent Se moieties can form diselenide bonds, making Se metals a good choice for constructing SCN. As shown in Figure 6B, Park group^[^ ^47^ ^]^ developed a diselenide‐crosslinked polymeric micelle for DOX delivery to treat prostate cancer. They synthesized a selenol‐bearing triblock copolymer that can self‐assemble and effectively encapsulate DOX into the hydrophobic core. Upon forming the nanostructures, the build‐in crosslinkers of the diselenide bond maintained the structural integrity of the nanomedicine in the physiological condition but responsively released the payloads in the ROS‐rich microenvironment, such as ample concentration of hydrogen peroxide. The crosslinked nanomedicine delivered significantly more DOX to the tumors, which are 1.69‐fold and 3.73‐fold higher than their non‐crosslinked counterparts and free drug, respectively, thus resulting in a highly efficacious anti‐cancer effect.

Calcium ions can attach phosphate or carboxyl groups through electrostatic force and attract significant attention in nanomedicine development because of their excellent biodegradability and biocompatibility. To develop nanomedicine with robust stability and responsive drug‐releasing capacity, Sun group^[^ ^48^ ^]^ synthesized an amphiphilic polypeptide‐based block copolymer (poly(glutamic acid), PGA). The PGAs assembled into nanoparticles, in which the γ‐carboxyl groups on PGA were crosslinked by calcium ions via electrostatic interaction and resulted in a calcium crosslinked nanomedicine (Figure 6C). The nanomedicine efficiently delivered the monocyte colony‐stimulating factor (M‐CSF) to the tumor site and responsively released it in acidic TME. The breakage of the calcium crosslinkers also consumed protons in TME, thus mitigating the acidic TME in tumors. The M‐CSF significantly inhibited tumor progress by inducing T‐cell mediated immunotherapy and reversing the immunosuppressive TME.

### 2.4 Built‐in SCNs constructed by other crosslinking strategies

Many other crosslinkers, including these forced by electrostatic interaction,^[^ ^49^ ^]^ particular enzymes,^[^ ^50^ ^]^ temperature‐triggered interaction,^[^ ^51^ ^]^ host–guest interaction,^[^ ^52^ ^]^ etc.,^[^ ^53^ ^]^ were employed in the construction of the SCNs with built‐in crosslinkers. These SCNs showed similar advantages, such as robust stability and responsive drug release, more effective drug loading, etc., like the nanomedicine mentioned above. They also exhibited unique properties, such as more versatile responsiveness, unique synthetic, and assembly processes, etc.

## 3 ON‐SURFACE CROSSLINKING STRATEGY

The clinical applications of nanomedicine, such as Doxil (liposomal DOX) and Abraxane (albumin‐bound paclitaxel), improve the biocompatibility of chemotherapy. However, these nanomedicines only contribute to marginal efficacy improvement over the conventional treatment. As depicted in CAPIR cascade above, various biological barriers, such as blood sheer force, blood proteins, renal clearance, dense extracellular matrix, high interstitial pressures of tumor tissue, cell uptake, intracellular drug release, etc., hinder the efficacy of nanomedicine toward cancer treatment.^[^ ^17^ ^]^ Sophisticated and well‐engineered nanomedicines are desirable to overcome these barriers simultaneously, as some contradictions emerge when we try to pass these biological barriers simultaneously. These contradictions are i) robust nanomedicines can stand the blood sheer force; however, overly stabilized nanomedicines may hinder the effective drug release. If nanomedicine is designed with marginal stability which is beneficial to drug release, they may not be able to bear the sheer force; ii) negatively charged nanomedicine can minimize opsonization^[^ ^20^ ^]^; however, the negative surface is not beneficial for the cellular uptake. If the nanomedicines are designed with a strongly positive charge, they have a large chance to experience opsonization and show poor PKs; iii) nanomedicines with larger sizes can escape the rapid renal clearance; however, the larger size hampers the tumor penetration of the nanomedicines. If the nanoparticles are designed with a smaller size (< 5 nm),^[^ ^54^ ^]^ they are vulnerable to renal clearance, most of them may not even reach the tumor sites.

**FIGURE 7:**
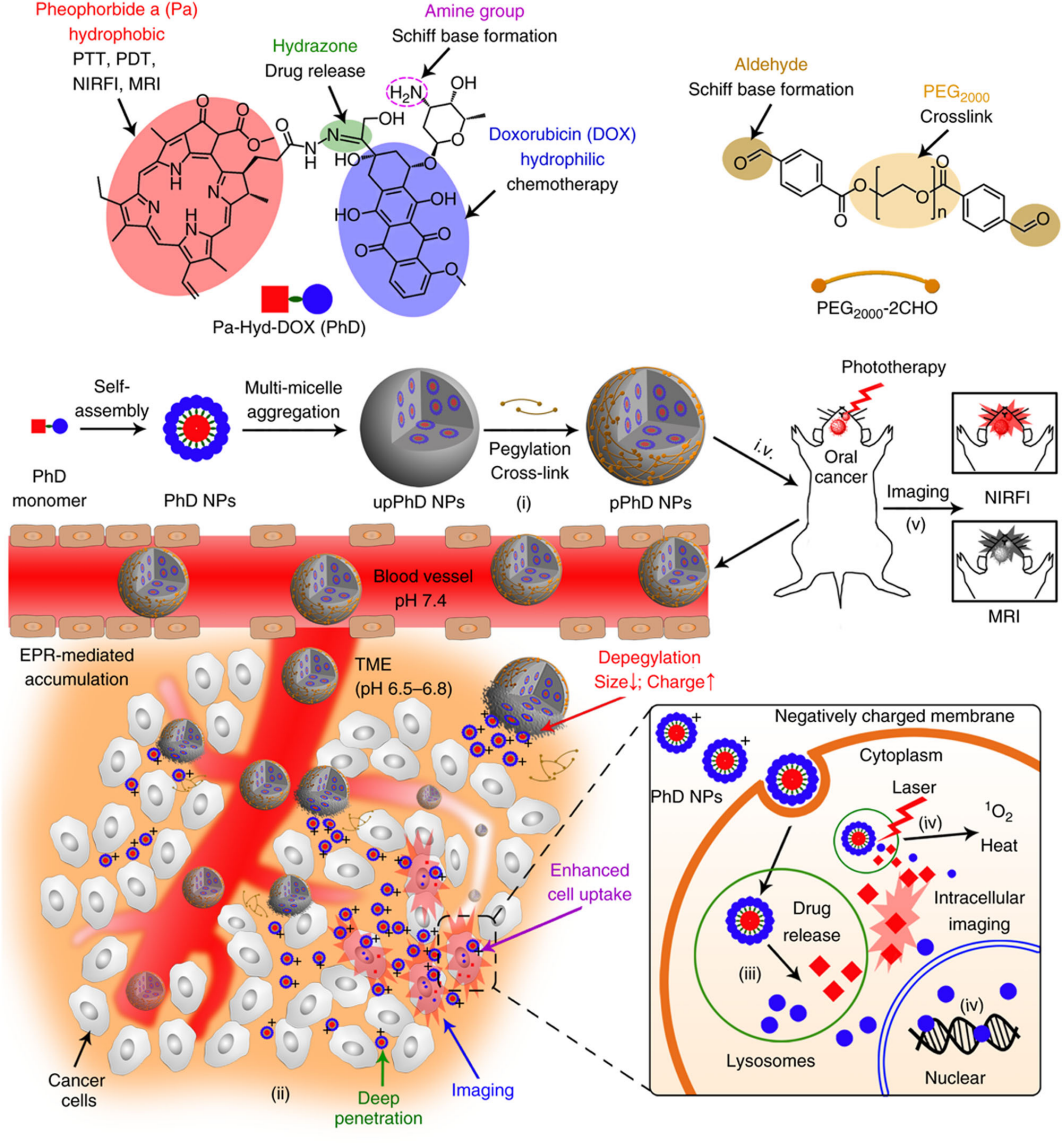
On‐surface crosslinking strategy to enhance the drug delivery efficiency. The on‐surface crosslinking strategy endows size transformation for the deep penetration and charge transformation for elevated cellular uptake to the nanomedicine. Reprinted with permission.^[^ ^55^ ^]^ Copyright 2018, Nature Publishing Group

SCNs with “on‐surface” crosslinkers are built to achieve robust stability and responsive drug‐releasing properties like the “built‐in” SCNs. Not only that, they exhibit many unique properties, such as size and surface charge transformation that can balance the aforementioned contradicts during the drug delivery. As shown in Figure 7, our lab developed a Trojan Horse nanotheranostic based on the “on‐surface” crosslinking strategy.^[^ ^55^ ^]^ Pheophorbide a (Pa) and DOX were conjugated through a hydrazone bond to form an amphiphilic prodrug (PhD). The PhDs self‐assembled into micelle‐like nanoparticles, then further grew to large nanoparticle by multi‐micelle aggregation. The PhD NPs present amine groups (from DOX) on the surface, thus exhibiting a strongly positive charge (42 mV), making the nanoparticle quite vulnerable to opsonization. We introduced a dialdehyde functionalized PEG_2000_ to react with amine moieties by forming the Schiff base. The PEGylation step can shield the strongly positive charge of the PhD NPs. Thanks to this “on‐surface” PEGylated crosslinking strategy, pPhD NPs remained weak surface charge and relatively large particle size (79 nm) during the blood circulation, which can eliminate the opsonization induced by strongly positive charge and rapid renal clearance caused by small particle size. While the pPhD NPs entered the TME, the slightly acidic pH cleaved the Schiff base and detached the PEGylated crosslinkers. In this way, pPhD NPs realized size and charge transformations. The particle size changed from large (79 nm) to ultrasmall (4 nm), and the surface charge rebounded to 35 mV. The ultrasmall size facilitated the nanoparticles to penetrate further in the dense tumor extracellular matrix, and the strongly positive charge substantially elevated the cellular uptake as the positive particles tend to adhere to the negatively charged cell membranes.^[^ ^56^ ^]^ This “on‐surface” SCN balanced the contradictions during the drug delivery and overcame the biological barriers. While the large size and weaker surface charge respectively hamper the tumor penetration and cell uptake, our “on‐surface” SCN transformed into a smaller size and strong surface charge, promoting tissue penetration and cellular uptake. The “on‐surface” crosslinking strategy also endows this nanomedicine with robust stability in serum and effective drug release in the tumor site. The hydrazone bonds can be readily cleaved in the lysosome (pH 5.0) and effectively release Pa and DOX. Owing to this intelligent on‐surface crosslinking strategy, the pPhD NPs can deliver the photosensitizer and drugs in a highly efficient manner, achieving a 100% complete cure rate on both orthotopic oral cancer and bladder cancer^[^ ^57^ ^]^ by the synergistic effect between phototherapy and chemotherapy.

**FIGURE 8:**
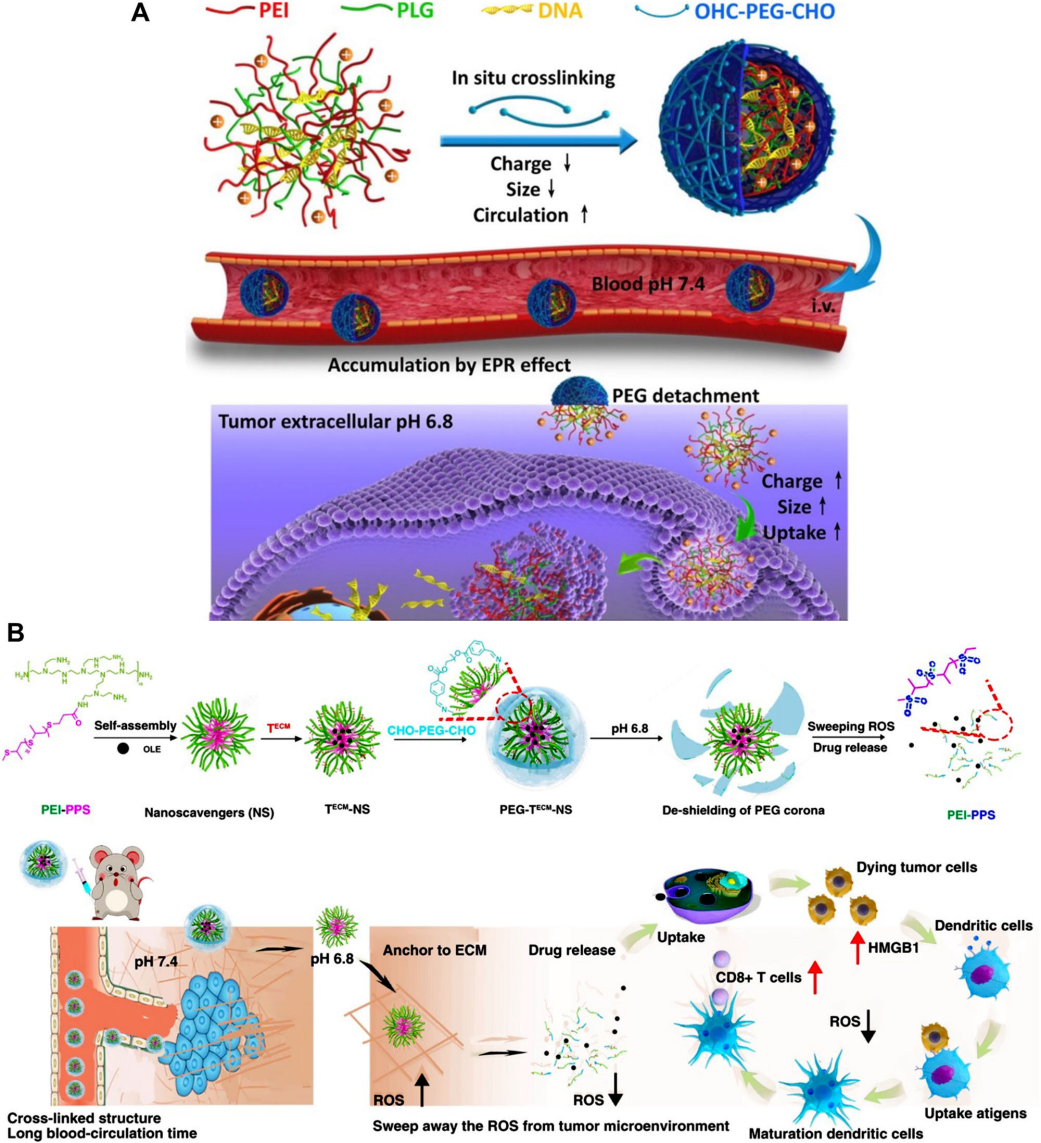
SCN with on‐surface crosslinkers. (A) SCN with on‐surface crosslinkers employed for gene delivery. Reprinted with permission.^[^ ^58^ ^]^ Copyright 2016, American Chemistry Society. (B) On‐surface type SCN employed for cancer immunotherapy. Reprinted with permission.^[^ ^59^ ^]^ Copyright 2020, Nature Publishing Group

Chen group developed a gene delivery system with on‐surface crosslinkers to overcome a series of biological barriers.^[^ ^58^ ^]^ As shown in Figure 8A, the delivery system was first assembled by polyethyleneimine (PEI) and poly‐l‐glutamate (PLG) to carry a plasmid DNA (pDNA) which expressed small hairpin RNA (shRNA) that targeted vascular endothelial growth factor (VEGF) to execute anti‐cancer effect. The resultant gene delivery system exposed many amine groups (from PEI) on the surface. The authors synthesized a dialdehyde terminated PEG to crosslink the nanomedicine on the surface by forming the Schiff base. The PEG crosslinkers can stabilize the nanoparticle and shield the positive charge from amine groups, thus decreasing the surface charge of the nanomedicine. The nanoparticle with shielded surface charge can reduce the opsonization and prolong blood circulation. The surface charge would be increased upon arriving at TME, as the slightly acidic pH can detach the surface PEG crosslinkers. The increased charge was beneficial to the cell uptake, as the cell membrane is negatively charged. This on‐surface crosslinking strategy can readily facilitate the drug delivery to tumors, which can significantly improve the anti‐tumor effect of the pDNA. The elevated reactive oxygen species (ROS) in the TME can weaken the immune response of the immunotherapy. To modulate the level o extracellular ROS, Chen group developed SCNs with on‐surface crosslinkers by using the same crosslinker as described above (Figure 8B).^[^ ^59^ ^]^ They synthesized a polymer comprised of PEI and PPS, which can assemble into nanomedicine with excessively exposed amine groups on the surface. Then, the dialdehyde terminated PEGs were introduced to crosslink the nanomedicine on the surface. Upon arrival at TME, the PEG crosslinkers were detached and released the PEI‐PPS, which can sweep the ROS production and release the loaded ICD inducer (oleandrin, OLE) to elicit the immunotherapeutic effect. This SCN can controllably release the therapeutics in the TME by the pH‐responsive crosslinkers to scavenge the ROS and modulate the immunosuppressive TME. Combining with ICD inducer, the SCNs effectively elicit the anti‐tumor immunity and increase the infiltration of the T lymphocytes, thus retreating the breast cancer progression on a mouse model.

## 4 INTER‐PARTICLE CROSSLINKING STRATEGY

SCN with “inter‐particle” crosslinkers are a multiparticle complex, in which many single nanoparticles unit together to form a relatively larger nanoparticle. The “inter‐particle” crosslinking strategy endows nanomedicine with advantages like controllable particle size and diversity in functionalities.

### 4.1 Inter‐particle crosslinking strategy for dendrimer‐based SCN

**FIGURE 9:**
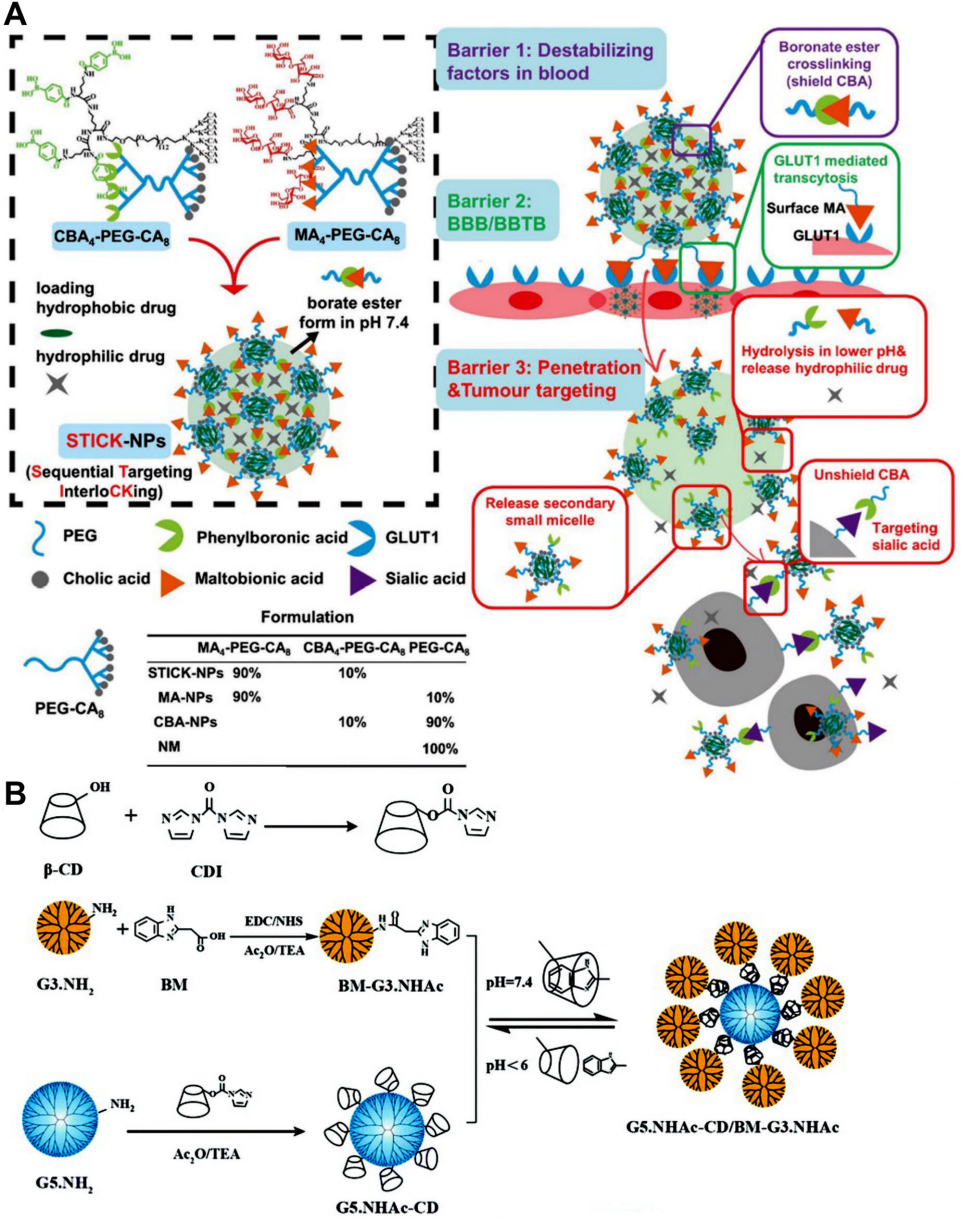
Organic SCNs constructed by inter‐particle crosslinking strategy. (A) The inter‐particle crosslinking strategy tethered different telodendrimer‐based nanoparticles by boronic ester bond for subsequential targeting to brain tumor. This SCN realizes size transformation for deep penetration and targeting moiety transformation for effective drug delivery to a brain tumor. Adapted with permission.^[^ ^60^ ^]^ Copyright 2020, Wiley‐VCH. (B) The inter‐particle crosslinking strategy tethered different dendrimers by guest–host interaction for improved tumor tissue penetration and anti‐cancer efficacy. Reprinted with permission.^[^ ^62^ ^]^ Copyright 2019, Royal Society of Chemistry

Patients with brain tumors generally show dismal prognosis, as a series of biological barriers hinder the therapeutic drugs from being delivered to brain tumor cells. Firstly, the blood sheer force destabilizes the nanomedicines, which may lead to premature drug release. Secondly, the blood‐brain barrier/blood‐brain tumor barrier (BBB/ BBTB) hampered therapeutics entering the brain area. Thirdly, cellular uptake is also a barrier set by the tumor cell membrane, even for some drugs that are able to penetrate the BBB/BBTB. Our lab developed “inter‐particle” SCNs by crosslinking multiple small micelles together to achieve better drug delivery to brain tumors. In the “inter‐particle” SCN, a sequential targeting process was realized to circumvent these critical physiological barriers and extensively improve the drug delivery efficiency to the brain tumor cells.^[^ ^60^ ^]^ As shown in Figure 9A, this “inter‐particle” SCN was composed of multiple hybrid polymeric micelles with maltobionic acid (MA) and 4‐carboxyphenylboronic acid (CBA) on the surface. These hybrid micelles can be crosslinked by forming the boronic ester bond between the MA and CBA. This unique crosslinking strategy allows the nanomedicine to experience size and target moiety transformations and sequentially to overcome the biological barriers to brain tumors. The “inter‐particle” SCN can stand the blood sheer force during the blood circulation, leading to a 5.4‐time higher AUC compared with conventional polymeric nanomedicine and 14‐times higher AUC than the free drugs. The surface MA mediated the nanomedicine to pass the BBB/BBTB via glucose‐transporter‐mediated transcytosis. In the acidic TME, the crosslinkers were broken down and then the transformation in size and targeting moiety was triggered. The larger nanomedicine collapsed into smaller ones which can realize deep penetration in the tumor site. Once broken down into tiny hybrid polymeric micelles, the exposed CBA can recognize the overexpressed sialic acid on the surface of the tumor cells and substantially improve the cellular uptake of nanoparticles into the brain tumor cells. Owing to these subsequential transformations, the “inter‐particle” SCN can significantly and safely inhibit tumor development and prolong the overall survival rate on the mouse model with aggregative and chemo‐resistant diffuse intrinsic pontine glioma. This formulation tackles multiple physiological barriers on‐demand with a simple and intelligent “inter‐particle” crosslinking design. Therefore, these features allow the “inter‐particle” SCN to unleash the potential of brain tumor therapeutics to improve their treatment efficacy. Inter‐particle crosslinking strategy can unit different small nanoparticles into a relatively larger one, endowing nanomedicine with robust serum stability and responsive size changes at the tumor site. With this strategy, Shi lab developed a series of dendrimer‐based SCN which exhibited several unique features.^[^ ^61^ ^]^ As shown in Figure 9B, they developed an inter‐particle SCN (G5.NHAc‐CD/BM‐G3) by employing a G5 dendrimer as core, and crosslinked multiple G3‐dendrimer to the core via “host–guest” interaction between cyclodextrin (CD) and benzimidazole (BM).^[^ ^62^ ^]^ The G5.NHAc‐CD/BM‐G3 can efficiently load DOX and exhibit an excellent pH‐responsive drug‐releasing profile. Compared to the non‐responsive crosslinked control SCN, the G5.NHAc‐CD/BM‐G3 showed better anti‐tumor efficacy and deeper penetration in Hela cell spheroid.

### 4.2 Inter‐particle crosslinking strategy for inorganic SCNs

**FIGURE 10:**
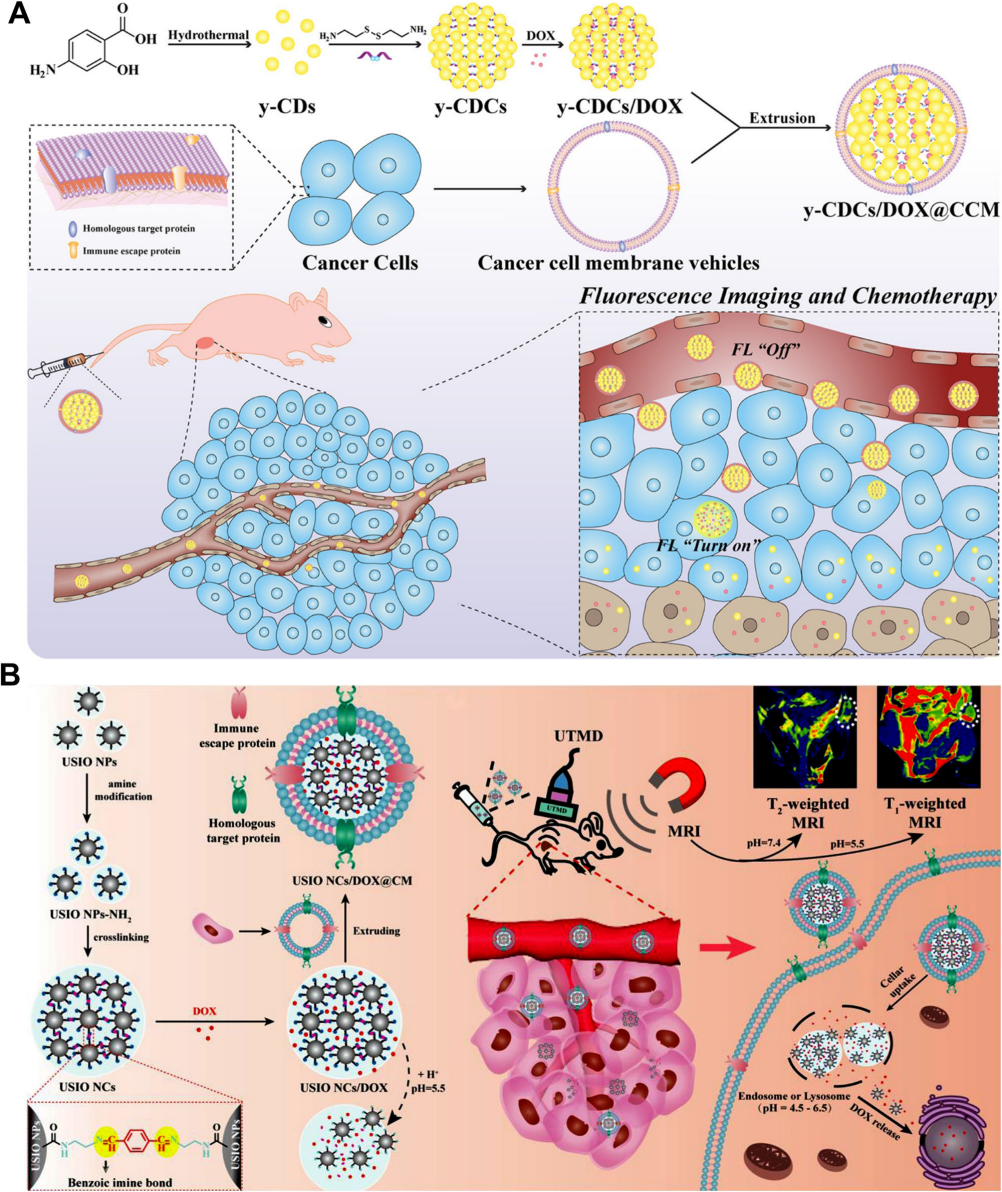
Inorganic SCNs constructed by inter‐particle crosslinking strategy. (A) The inter‐particle crosslinking strategy tethered carbon dots (CDs) by disulfide bond for doxorubicin delivery. Reprinted with permission.^[^ ^64^ ^]^ Copyright 2021, American Chemistry Society. (B) The inter‐particle crosslinking strategy tethered iron oxide nanoparticles by benzoic imine bond for switchable T1/T2 MRI and doxorubicin delivery. Reprinted with permission.^[^ ^66^ ^]^ Copyright 2021, Elsevier

Inorganic nanoparticles, such as carbon dots, iron oxide nanoparticles, and quantum dots, exhibit excellent imaging capacities.^[^ ^63^ ^]^ To improve imaging specificity and obtain more accurate functional and molecular information at the tumor site, imaging approaches with tunable signals that respond to particular stimuli are desirable. Shi lab employed an inter‐particle crosslinking strategy to unit a cluster of inorganic nanoparticles to achieve imaging responsiveness and efficient drug release. As shown in Figure 10A, they developed a redox‐responsive, DOX‐loaded yellow fluorescent carbon dot SCN (y‐CDC) covered by membranes from cancer and red‐blood cells, endowing nanomedicine with antifouling properties, immune escape ability to reduce macrophage uptake, and homologous targeting capability against B16 melanoma cells.^[^ ^64^ ^]^ The formulated y‐CDCs can load high‐content DOX (81% w/w), which can responsively be released in the GSH‐abundant compartment in the tumor cells. Moreover, the fluorescence of carbon dots in y‐CDCs was quenched when the nanoparticles were crosslinked and can be recovered when the drug was released. On the B16 xenograft mouse model, the resultant nanomedicine showed deeper tumor tissue penetration and better anti‐tumor efficacy than the GSH‐insensitive counterpart. Iron oxide nanoparticles (IO NPs) show excellent MRI properties and their MRI properties can be changed by varying the particle size. The relatively larger IO NPs (> 5 nm) generally showed more T2 MR signal, and comparatively, ultras‐small IO NPs (< 5 nm) behave like T1 MRI contrast agents.^[^ ^65^ ^]^ Based on this mechanism, Shi lab crosslinked multiple 3.2 nm ultrasmall iron oxide nanoparticles (USIO NPs) by using an inter‐particle crosslinking strategy (Figure 10B).^[^ ^66^ ^]^ The USIO NPs were crosslinked by pH‐responsive benzoic imine bonds and loaded with anti‐cancer drug doxorubicin (DOX). The crosslinked USIO NPs showed strong T2 MRI signals, as they aggregated together to behave like large IO NPs. Once arrived at the acidic TME, the crosslinked USIO NPs responsively disassembled in acidic pH to release the DOX and USIO NPs and realize dynamic T2/T1 switchable signals. Further, the dynamic MRI and tumor chemotherapy can be further enhanced through the ultrasound‐induced sonoporation effect. Shi lab also employed cystamine‐mediated inter‐particle crosslinkers to tether 3.3 nm USIO NPs to form a GSH‐responsive SCN.^[^ ^67^ ^]^ The SCN realized sensitive switchable T2/T1‐weighted dual‐mode MR imaging on the breast cancer mouse model.

Furthermore, the “inter‐particle” crosslinking strategy has been widely explored in other fields, such as “inter‐particle” crosslinked hydrogel with shear‐thinning and self‐healing properties,^[^ ^68^ ^]^ gold nanoparticles^[^ ^69^ ^]^ were “inter‐particle” crosslinked for high‐sensitive detection based on their unique surface plasmon resonance (SPR) effect.

The categories of the SCNs discussed in this review are summarized in Table 1, including their cleavable crosslinker, stimuli, dynamic responses, benefits, and the corresponding references. The SCNs are mainly classified into three categories based on the crosslinking strategies, including built‐in, on‐surface, and inter‐particle crosslinking nanomedicines. The SCNs can be built with different cleavable crosslinkages, such as disulfide bond, boronic ester, hydrazone bond, orthoester bond, iron‐catechol coordination, diselenide‐bond, calcium‐mediated electrostatic force, Schiff base, host–guest interaction, benzoic imine bond, temperature‐sensitive bond, enzyme cleavable bond. The cleavable bonds make the SCNs responsive to different stimuli in TME, such as redox, acidosis, temperature, temperature, and enzyme, and activate a series of responses of the SCNs. With these unique designs, the SCNs exhibit myriads benefits in the fields of cancer therapy and diagnosis; SCNs can extensively load more drugs, overcome different biological barriers, specific targeting, and subsequently improve the anti‐tumor efficacy and imaging capacities.

**TABLE 1:** Summary of the three categories of SCNs

Crosslinking strategies	Crosslinkers	Stimuli	Responses	Benefits	Refs.
Built‐in	Disulfide bond	GSH	Changes in structural integrity	Improved efficacy and imaging	[24g]
	Disulfide bond	GSH	Changes in structural integrity	“All‐in‐one” imaging and improved in vivo performance	[24h]
	Disulfide bond	GSH	Changes in structural integrity; dual T1/T2 “OFF” to “ON”	Improved in vivo performance; Exceptional MRI sensitivity and detection limit	[24i]
	Disulfide bond	GSH	Changes in structural integrity	Improved efficacy	[26, 28]
	Boronic ester	pH	Changes in structural integrity	High drug loading; improved in vivo performance	[40]
	Ester bond	pH	Changes in structural integrity	High drug loading; improved in vivo performance	[41]
	Hydrazone bond	pH	Changes in structural integrity	High drug loading; improve in vivo performance	[42]
	Orthoester bond	pH	Changes in structural integrity	Improved in vivo performance	[43]
	Iron‐catechol coordination	pH	Changes in structural integrity	High drug loading; robust stability; improved in vivo performance	[45]
	Diselenide‐bond	Reactive oxygen species	Changes in structural integrity	Improved in vivo performance	[47]
	Electrostatic force	pH	Changes in structural integrity	Improved in vivo performance	[49, 50]
	Other responsive bonds	Enzyme; temperature; pH; etc.	Changes in structural integrity	Improved in vivo performance	[51, 52, 53, 54]
On‐surface	Schiff‐base	pH	Changes in size, surface charge	Deeper tissue penetration; elevated cell uptake and efficient drug delivery	[56, 58]
	Schiff‐base	pH	Changes in surface charge	Elevated cell uptake; overcome different biological barriers	[58]
	Schiff‐base	pH	Changes in structural integrity	Improved immune responsiveness	[59]
Inter‐particle	Boronic ester	pH	Size changes	Sequential tumor targeting; circumvent physiological barriers	[60]
	Cylodextran‐benzimidazole host–guest interaction	pH	Changes in structural integrity	Deeper penetration; improved efficacy	[62]
	Disulfide bond	GSH	Fluorogenic property, size changes	High drug loading; better fluorescence imaging and improved efficacy	[64]
	Benzoic imine bond	pH	Switchable T2 to T1 MRI	Better imaging; improved efficacy	[66]

## 5 CONCLUSION AND PERSPECTIVES

Crosslinked nanomedicines have been widely developed for drug delivery, diagnosis, and imaging. This review highlights the recent advances of the stimuli‐responsive crosslinked nanomedicines (SCNs), which are constructed with different crosslinking strategies. We classified the crosslinking approaches into three categories, including built‐in, on‐surface, and inter‐particle crosslinking strategies. These strategies endow the SCNs with robust serum stability and stimuli‐responsive payloads release, which can notably prevent premature drug release, prolong the blood circulation, and control the drug release at targeting locale. Furthermore, the crosslinking strategies also endow the nanomedicine with the capabilities to trigger a series of transformations along with the cleavage of crosslinkers, such as the unique transformations in size, surface charge, targeting moiety, nanostructural integrity, and imaging signal. These transformations can accommodate the complicated biological conditions and bring various advantages to cancer therapy and diagnosis. For instance, these can be used to extensively improve the drug delivery efficiency by its unique size and charge transformations, mediate subsequential tumor targeting by the surface moiety transformation, and precisely detect tiny tumors by the integrity and imaging signal transformation.

The stimuli‐crosslinking strategy shows promising potential for cancer treatments. However, SCNs also have a large room to be improved due to the following reasons: i) SCNs are complicated and personalized, which need sophisticated design and complex chemistry to meet different cancer‐treating requirements. ii) The currently developed SCNs mainly focus on the improvements in PKs and controllable drug release. The tissue penetration that directly affects drug concentration in tumors is not often considered. iii) The SCNs generally exhibit an excellent sustained drug‐releasing pattern; however, some SCNs built with blunt crosslinkers may not be able to release the drug efficiently. For future development, the SCNs can be improved in aspects of more specific factors, including i) integration of different transformations in one SCN may lead to the development of more powerful nanomedicine for cancer treatments. ii) Customization of the transformation based on the unique cancer biology of particular cancer types may endow the SCNs with more anti‐cancer powers. iii) The design of crosslinkers to consume particular signaling molecules in the TME during cleavage may remodel the TME and provide valuable therapeutic functions, such as immune modulation and apoptosis, to further improve the anti‐cancer efficacy.

## CONFLICT OF INTEREST

The authors declare no conflict of interest.
